# Clinical and Basic Research on Dopa-Responsive Dystonia: Neuropathological and Neurochemical Findings

**DOI:** 10.14789/ejmj.JMJ24-0023-R

**Published:** 2025-01-30

**Authors:** YOSHIAKI FURUKAWA

**Affiliations:** 1 Department of Neurology, Juntendo Tokyo Koto Geriatric Medical Center, Tokyo, Japan; 1 Department of Neurology, Juntendo Tokyo Koto Geriatric Medical Center, Tokyo, Japan; 2 Department of Neurology, Juntendo University Graduate School of Medicine, Tokyo, Japan; 2 Department of Neurology, Juntendo University Graduate School of Medicine, Tokyo, Japan

**Keywords:** dopa-responsive dystonia (DYT5a and DYT5b), GTP cyclohydrolase 1, tetrahydrobiopterin, tyrosine and tryptophan hydroxylases, striatal dopamine

## Abstract

Dopa-responsive dystonia (DRD) is a clinical syndrome characterized by childhood-onset dystonia and a dramatic and sustained response to low doses of levodopa. Typically, DRD presents with gait disturbance due to foot dystonia, later development of parkinsonism, and diurnal fluctuation of symptoms. Since the discovery of mutations responsible for DRD in *GCH1*, coding for GTP cyclohydrolase 1 (GTPCH) that catalyzes the rate-limiting step in tetrahydrobiopterin (BH_4_: the cofactor for tyrosine hydroxylase [TH]) biosynthesis, and in *TH*, coding for TH in catecholamine biosynthesis, our understanding of this syndrome has greatly increased. However, the underlying mechanisms of phenotypic heterogeneity are still unknown and physicians should learn from genetic, pathological, and biochemical findings of DRD. Neuropathological studies have shown a normal population of cells with decreased melanin and no Lewy bodies in the substantia nigra of classic GTPCH-deficient and TH-deficient DRD. Neurochemical investigations in GTPCH-deficient DRD have indicated that dopamine reduction in the striatum is caused not only by decreased TH activity resulting from low cofactor content but also by actual loss of TH protein without nerve terminal loss. This striatal TH protein loss may be due to a diminished regulatory effect of BH_4_ on stability of TH molecules. Neurochemical findings in an asymptomatic *GCH1* mutation carrier versus symptomatic cases suggest that there may be additional genetic and/or environmental factors modulating the regulatory BH_4_ effect on TH stability and that the extent of striatal protein loss in TH (rather than that in GTPCH) may be critical in determining the symptomatic state of GTPCH-deficient DRD.

## Introduction

Dopa-responsive dystonia (DRD) is a clinical syndrome characterized by childhood-onset dystonia and a dramatic and sustained response to relatively low doses of oral administration of levodopa^1-3)^. This syndrome typically presents with gait disturbance resulting from foot dystonia, later development of some parkinsonian features, and diurnal fluctuation of symptoms (worsening of symptoms toward the evening and alleviation of symptoms in the morning after sleep). In 1971, Segawa et al.^4)^ and Castaigne et al.^5)^ independently reported clinical details of one family each with DRD, for which they employed the following terms at that time, “hereditary progressive basal ganglia disease with marked fluctuation” and “progressive extrapyramidal disorder,” respectively. Advances in the genetics and biochemistry of DRD^2, 3, 6-14)^ have shown that the former had autosomal dominant GTP cyclohydrolase 1 (GTPCH) deficiency (DYT5a^15)^) and the latter had autosomal recessive tyrosine hydroxylase (TH) deficiency (DYT5b^16)^). This short review summarizes neuropathological features of classic DRD and neurochemical characteristics of symptomatic and asymptomatic cases with GTPCH-deficient DRD^8, 38-42)^, the most common form of DRD.

## Summarized clinical features and genetics of classic DRD

**Figure 1:**
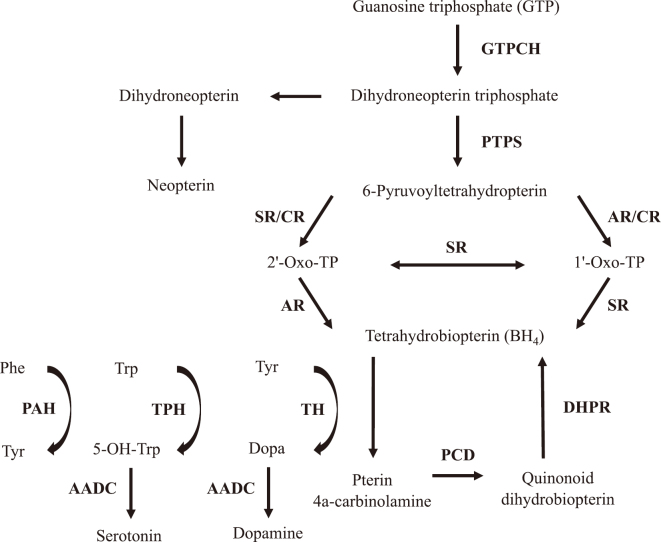
Biosynthesis and regeneration of tetrahydrobiopterin (BH_4_) and BH_4_-dependent hydroxylation of aromatic amino acids AADC = aromatic amino acid decarboxylase; AR = aldose reductase; CR = carbonyl reductase; DHPR = dihydropteridine reductase; GTPCH = GTP cyclohydrolase 1; PAH = phenylalanine hydroxylase; PCD = pterin-4a-carbinolamine dehydratase; Phe = phenylalanine; PTPS = 6-pyruvoyltetrahydropterin synthase; SR = sepiapterin reductase; TH = tyrosine hydroxylase; TPH = tryptophan hydroxylase; Trp = tryptophan; Tyr = tyrosine; 1'-Oxo-TP = 2'-hydroxy-1'-oxopropyltetrahydrobiopterin; 2'-Oxo-TP = 1'-hydroxy-2'-oxopropyltetrahydrobiopterin; and 5-OH-Trp = 5-hydroxytriptophan. Classic dopa-responsive dystonia (DRD) can be caused by autosomal dominant GTPCH deficiency (the most common form of DRD), by autosomal recessive TH deficiency, or rarely by autosomal recessive SR deficiency. Patients with autosomal recessive BH_4_-related enzyme deficiencies, including recessively inherited severe GTPCH deficiency, generally develop BH_4_-dependent hyperphenylalaninemia; an exception is autosomal recessive SR deficiency (in which BH_4_ is synthesized through the salvage pathway in peripheral tissues).

**Figure 2:**
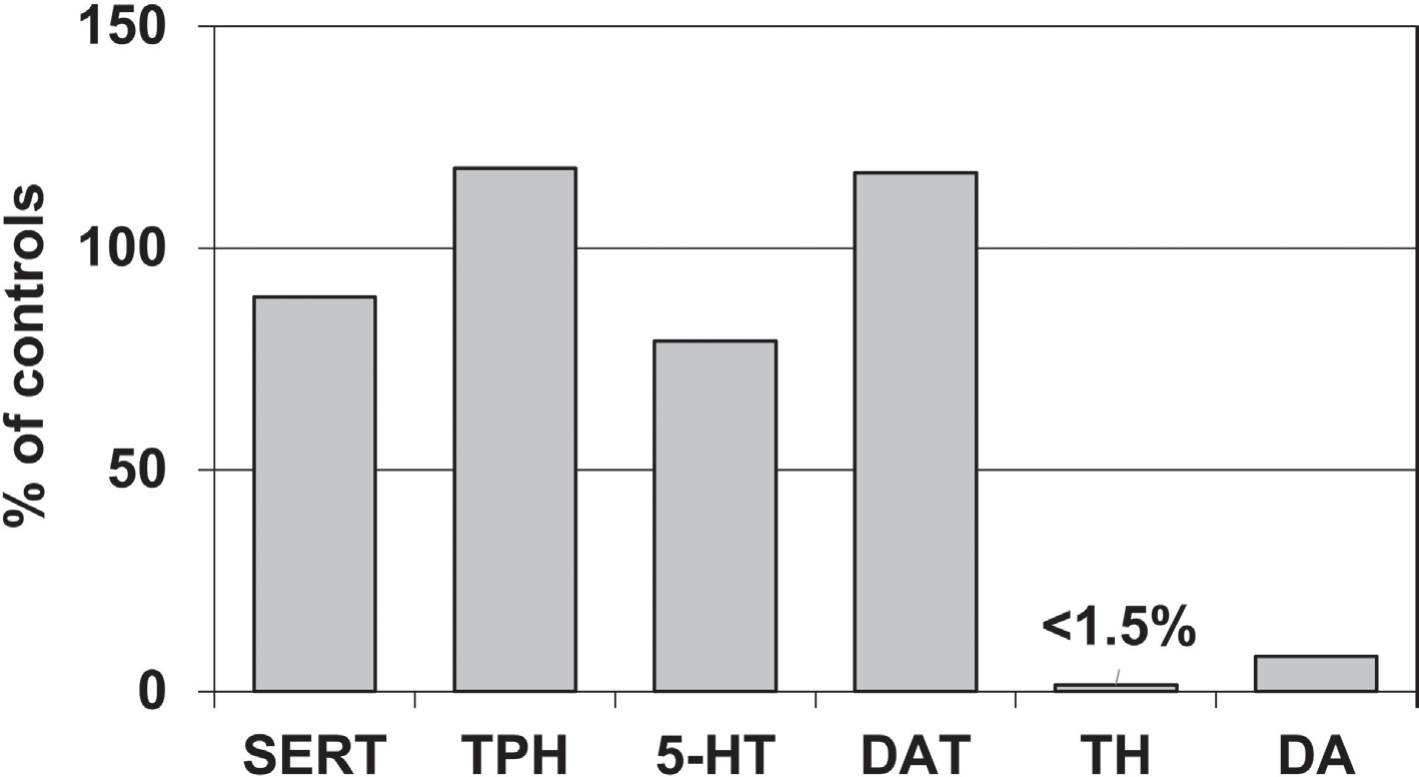
Levels of serotonin transporter (SERT), tryptophan hydroxylase (TPH), 5-hydroxytryptamine (5-HT = serotonin), dopamine transporter (DAT), tyrosine hydroxylase (TH), and dopamine (DA) in the putamen of a genetically confirmed patient with GTP cyclohydrolase 1-deficient dopa-responsive dystonia^3, 8, 38, 41, 42)^ (Case 1), expressed as percentages of age-matched control mean values (SERT, TPH, 5-HT, and DA data are from reference 41 and DAT and TH data are from reference 38). In Case 1, the TH protein concentration in the caudal subregion of the putamen was less than 1.5% of the mean value for controls.

For classic DRD (Table 1), there are three known causative genes: 1) the *GCH1* gene on chromosome 14q, encoding GTPCH, the rate-limiting enzyme in the biosynthetic pathway for tetrahydrobiopterin (BH_4_: the natural cofactor for TH, tryptophan hydroxylase [TPH], and phenylalanine hydroxylase) (Figure 1); 2) the *TH* gene on 11p, coding for TH, the rate-limiting enzyme in catecholamine biosynthesis; and 3) the *SPR* gene on 2p, encoding sepiapterin reductase (SR), an enzyme involved in the final step of BH_4_ synthesis^17)^. Many DRD patients have dominantly inherited *GCH1* variants (GTPCH- deficient DRD: the major form of DRD^14, 15)^) and relatively few DRD cases have recessively inherited *TH* variants (TH-deficient DRD: the mild form of TH deficiency^16, 18)^). Rarely, recessively inherited *SPR* mutations (leaky or partially penetrant splicing variants), which may result in only a slight reduction of SR activity, can also cause DRD (SR-deficient DRD: the very mild form of SR deficiency^17)^); most of cases with SR deficiency are known to develop more severe symptoms and signs (motor and speech delay, truncal hypotonia, cognitive impairment, psychiatric and behavioral problems, paroxysmal stiffening, etc.)^19, 20)^. Patients with other autosomal recessive BH_4_-deficient disorders generally develop BH_4_-dependent hyperphenylalaninemia^14, 19)^.

**Table 1:** Clinical characteristics of classic dopa-responsive dystonia (DRD)

1. Onset generally in childhood; early motor development is normal.
2. Onset of dystonia in a limb, typically foot dystonia resulting in gait disturbance.
3. Later development of some parkinsonian features; tremor is mainly postural.
4. Presence of brisk deep-tendon reflexes in the legs and/or the striatal toe in many patients.
5. Diurnal fluctuation of symptoms (aggravation of symptoms toward the evening and their alleviation in the morning after sleep); the degree of fluctuation is variable.
6. Gradual progression to generalized dystonia; typically, more pronounced dystonia in the legs throughout the disease course.
7. Attenuation in the magnitude of diurnal fluctuation with age and disease progression.
8. A dramatic and sustained response (complete or near-complete responsiveness of symptoms) to low doses of levodopa.
9. Maximum benefit is generally achieved by less than 300-400 mg/day of levodopa with a decarboxylase inhibitor.
10. Absence of motor adverse effects of chronic levodopa therapy (motor response fluctuations and levodopa-induced dyskinesias) under optimal doses of levodopa.
11. Female predominance of clinically affected individuals in GTPCH-deficient DRD.

GTPCH = GTP cyclohydrolase 1.

In GTPCH-deficient and TH-deficient DRD, wide variations in expressivity have been demonstrated but no correlations between specific clinical features and types of variants in *GCH1* and *TH* have been established^3, 12, 18, 21-36)^. As the pathogenesis of phenotypic heterogeneity in DRD (including gender-related incomplete penetrance in GTPCH-deficient DRD^12, 37)^) is still unknown and clinical suspicion is a key to the diagnosis of this treatable syndrome, physicians should learn from clinical and basic research findings of DRD and should know not only the typical phenotype but also the broad phenotypic spectrum of GTPCH-deficient and TH-deficient DRD; the clinical phenotype of genetically confirmed GTPCH-deficient DRD has been extended to include various types of focal dystonia (e.g., adult-onset guitarist's cramp), DRD simulating cerebral palsy or spastic paraplegia, adult-onset parkinsonism, and so forth^15, 17)^. In the case of TH deficiency, based on the severity of symptoms and responsiveness to levodopa, the clinical phenotypes are classified into TH-deficient DRD (mild form), TH-deficient infantile parkinsonism with motor delay (severe form), and TH-deficient progressive infantile encephalopathy (very severe form)^16, 17)^.

## Neuropathological characteristics of classic GTPCH-deficient and TH-deficient DRD

Pathological findings have been reported in four symptomatic female cases (age at death: Case 1; 19 years^3, 8, 38)^, Case 2; 68 years^38)^, Case 3; 77 years^43, 44)^, and Case 4; 90 years^45)^) with typical GTPCH-deficient DRD, one asymptomatic female case (55 years^39)^) with a *GCH1* variant (in an autosomal- dominant DRD family linked to the *GCH1* locus^7)^), and one male patient (49 years^31, 46)^) with typical TH-deficient DRD. In the substantia nigra (SN), neuropathological studies demonstrated a normal cell count without gliosis and no Lewy bodies in all of the symptomatic and asymptomatic cases with GTPCH-deficient DRD. There were no degenerative changes also in other brain areas of these cases. Characteristically, however, the number of melanin-containing cells was markedly decreased in the SN of the *GCH1*-associated symptomatic and asymptomatic cases^8, 38, 39, 43)^, except for the oldest patient (Case 4)^45)^. Schreglmann and colleagues^46)^ similarly found a striking pallor of melanin-containing neurons without cell loss and no evidence of Lewy body formation in the SN of the typical TH-deficient DRD patient. There have been no reports of pathological findings in SR-deficient DRD.

## Neurochemical features of symptomatic versus asymptomatic GTPCH-deficient DRD

Brain biochemical data from two of the four symptomatic cases with GTPCH-deficient DRD (Cases 1 and 2) and the asymptomatic *GCH1* mutation carrier are available^8, 38-42)^; consistent with the neuropathological findings (see above), striatal α-synuclein levels measured by quantitative blot immunolabeling^39, 47, 48)^ were normal in Cases 1 and 2. In the putamen, total biopterin (BP: BP includes BH_4_, quinonoid dihydrobiopterin, dihydrobiopterin, and oxidized biopterin, and most of brain BP exists as BH_4_^13, 49, 50)^) and total neopterin (NP: NP consists of degradation products [dihydroneopterin and oxidized neopterin] of dihydroneopterin triphosphate^13, 51)^ [Figure 1]) levels were substantially lower in GTPCH-deficient DRD Cases 1 and 2 (mean: -84% and -62%) than in age-matched normal controls^38)^. Striatal subregional dopamine data pointed to an involvement of the caudal portion of the putamen as the striatal subregion that was most affected by dopamine loss in these patients (-88%)^8, 38)^. It is well known that this striatal subdivision is most affected by loss of dopamine in patients with Parkinson's disease (PD)^52-54)^. Dopamine content in the caudal putamen was found to be normal in an autopsied patient with DYT1 dystonia^55)^. In the *GCH1*-associated asymptomatic case, decreases of BP and NP concentrations in the putamen (-82% and -57%) paralleled those in the two symptomatic cases^39)^. However, in this asymptomatic *GCH1* mutation carrier, dopamine content in the caudal subdivision of the putamen was not as severely reduced (-44%) as in the symptomatic cases. Consistent with other postmortem data indicating that greater than 60-80% of striatal dopamine loss is necessary for overt motor symptoms to occur^52)^, the maximal 44% dopamine reduction in the striatum of the asymptomatic *GCH1* mutation carrier was not sufficient to produce any symptoms of GTPCH-deficient DRD.

In contrast to PD patients, striatal levels of aromatic amino acid decarboxylase (AADC), dopamine transporter (DAT), and vesicular monoamine transporter 2 were normal in the two symptomatic cases with GTPCH-deficient DRD (Cases 1 and 2), indicating that dopaminergic terminals in the striatum are preserved in this disorder^38)^; an age- related decline of putaminal BP during adulthood could contribute to late-onset parkinsonism recognized in Case 2^35, 56, 57)^. However, TH protein concentrations were markedly decreased in the putamen of GTPCH-deficient DRD Cases 1 and 2 (> -97%)^38)^. These findings have suggested that striatal dopamine reduction in GTPCH-deficient DRD is caused not only by decreased TH activity resulting from low cofactor content but also by actual loss of TH protein without nerve terminal loss. The human brain data are compatible with TH protein loss but preserved AADC in brains of BH_4_-deficient mice^58)^. Even in zebrafish *gch1*^-/-^ mutants, there was no loss of ascending dopaminergic neurons, whereas TH protein levels were decreased^59)^. In contrast to the symptomatic cases, TH protein content in the putamen was only moderately reduced in the asymptomatic *GCH1* mutation carrier (-52%)^39)^. Striatal TH protein reduction in GTPCH-deficient DRD may be caused by a diminished regulatory effect of BH_4_ on the steady-state level (stability/expression) of TH molecules. Because TH protein levels in the SN, where striatal TH molecules are synthesized, were normal in both symptomatic cases^8, 38)^, BH_4_ could control stability rather than expression of this enzyme. This is supported by a report showing brain loss of TH protein but not of *TH* mRNA in BH_4_-deficient mice^58)^. Although there have been no reports of human neurochemical data in classic TH-deficient DRD, animal experiments have demonstrated normal number of TH-positive neurons in the SN and severely reduced TH immunostaining, associated with normal DAT staining, in the striatum of DRD *TH* knock-in mice^60, 61)^. Kawahata and colleagues^62)^ have demonstrated that increased TH phosphorylation (which is higher in the terminals than the soma) facilitates TH degradation and have suggested that not only an increase in TH phosphorylation but also additional factors are involved in TH protein loss in BH_4_/dopamine deficiencies.

The postmortem observations in GTPCH-deficient DRD Cases 1 and 2 are consistent with normal DAT and ^18^F-fluorodopa imaging in DRD cases (including Case 2) and adult-onset ‘benign (neurometabolic)' parkinsonian subjects from DRD families^63-69)^. In this case, parkinsonism appears to be caused by a defect in dopamine biosynthesis due to haploinsufficiency of *GCH1* and the nigrostriatal dopaminergic terminals are preserved^35)^. Patients with this type of parkinsonism respond markedly to low doses of levodopa and remain functionally normal for a long period of time without developing motor adverse effects of chronic levodopa therapy. Examinations of DAT and ^18^F-fluorodopa imaging revealed no abnormalities even in more severely affected patients with autosomal recessive SR deficiency^35)^. Rose and colleagues^61)^ demonstrated normal DAT and reduced TH immunostaining in the striatum of aged DRD *TH* knock-in mice with parkinsonism and have suggested that abnormal DAT imaging found in some adult-onset parkinsonian patients in DRD pedigrees^17, 70)^ could reflect homeostatic DAT downregulation. On the other hand, there was an adult-onset parkinsonian patient associated with a heterozygous *GCH1* variant, who showed abnormal DAT imaging and low BH_4_ and normal NP in cerebrospinal fluid (CSF)^70)^. This pattern of CSF and brain pterin changes (decreased BP and normal NP^11, 14, 35, 38)^) indicates that parkinsonism in the patient is caused by neurodegeneration. Although it has been previously hypothesized that chronic dopamine depletion in GTPCH-deficient DRD could directly predispose to nigral degeneration^70)^, recent data of zebrafish *gch1*^-/-^ mutants and aged DRD *TH* knock-in mice have not supported this hypothesis^59, 61)^. In fact, the autopsied patient with classic GTPCH-deficient DRD (Case 4), whose duration of illness was 82 years, demonstrated no degenerative changes in the SN, even though this case was not treated with levodopa for 43 years^45)^. Shin and colleagues^71)^ have recently indicated that *GCH1* variants can cause adult-onset parkinsonism by unmasking subclinical nigrostriatal dopaminergic degeneration due to incidental Lewy body disease.

**Figure 3:**
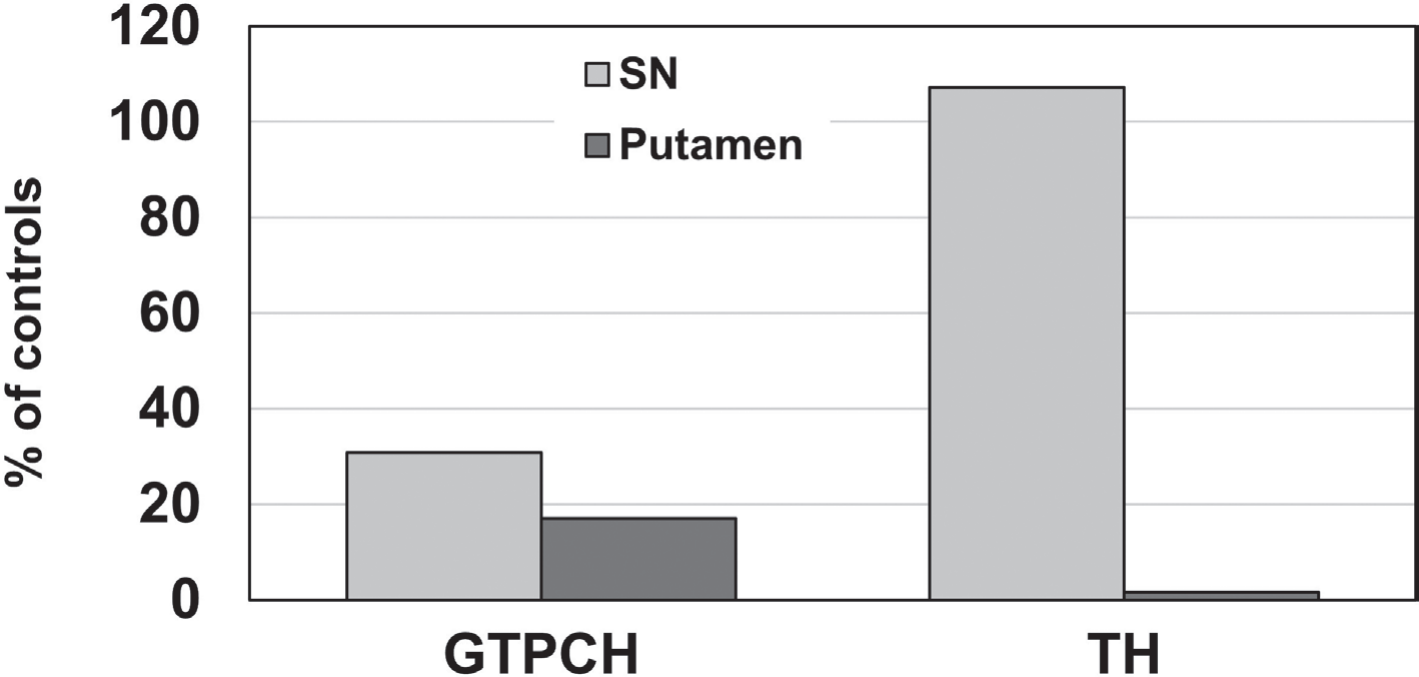
Levels of GTP cyclohydrolase 1 (GTPCH) and tyrosine hydroxylase (TH) in the substantia nigra (SN) and putamen of a genetically confirmed patient with GTPCH-deficient dopa-responsive dystonia^3, 8, 38, 41, 42)^ (Case 1), expressed as percentages of age-matched control mean values (GTPCH data are from reference 42 and TH data are from references 8 and 38). In Case 1, in contrast to the GTPCH protein concentrations (equally reduced in the SN and putamen), there was a marked difference in the TH protein levels between the SN and putamen.

As BH_4_ is also the cofactor for TPH, it has been assumed that partial BH_4_ deficiency in GTPCH- deficient DRD results in lowering of brain serotonin. However, all serotonin markers (serotonin [5-hydroxytryptamine]^72)^, TPH^54)^, and serotonin transporter^73)^) were found to be normal in the striatum of GTPCH-deficient DRD Case 1^41)^ (Figure 2). In marked contrast to the TH protein levels (normal in the SN and severely reduced in the putamen^8, 38)^), GTPCH protein concentrations were equally decreased in the SN and putamen of this symptomatic DRD case (-70% and -83%)^42)^ (Figure 3). Suzuki and colleagues^74)^ have found substantial loss of GTPCH protein in phytohemagglutinin-stimulated mononuclear blood cells from GTPCH-deficient DRD patients (with a frameshift or missense *GCH1* mutation) and have indicated that reduction of the amount of GTPCH protein, which is independent of the *GCH1* mutation type, contributes to the mechanism of dominant inheritance. In the asymptomatic *GCH1* mutation carrier, decreases of GTPCH protein levels in the SN and putamen (-58% and -74%) paralleled those in GTPCH-deficient DRD Case 1^42)^. Thus, in the putamen, consistent with BP and NP data^38, 39)^, GTPCH protein concentrations did not distinguish DRD Case 1 from the asymptomatic carrier. It has been reported that there was no difference of *GCH1* mRNA expression in phytohemagglutinin-stimulated mononuclear blood cells between symptomatic and asymptomatic GTPCH-deficient DRD family members^75)^. Moreover, although it is known that penetrance of *GCH1* mutations is higher in females than in males^12, 37)^, *GCH1* mRNA levels in human brain were not lower in females compared to males^76)^. Therefore, the genetic and biochemical data obtained so far suggest that the extent of putaminal loss in TH protein, rather than that in GTPCH protein, may be crucial for determining the symptomatic state of classic GTPCH-deficient DRD.

## Conclusion

In classic GTPCH-deficient and TH-deficient DRD, neuropathological investigations have demonstrated a normal cell count with reduced melanin and no evidence of Lewy body formation in the SN. Human and experimental (e.g., BH_4_-deficient mice, zebrafish *gch*^-/-^ mutants, and DRD *TH* knock-in mice) neurochemical data have indicated that dopamine reduction in the striatum of GTPCH-deficient DRD (the most common form of DRD) is caused not only by decreased TH activity owing to low cofactor (BH_4_) content but also by actual loss of TH protein without degeneration of nigrostriatal dopaminergic neurons. This striatal TH protein reduction may result from a diminished regulatory effect of BH_4_ on TH stability (consequent to congenital BH_4_ deficiency); thus, all of the abnormal gene products identified in classic DRD (GTPCH, TH, and SR [rare]) are related to striatal TH molecules. The different degrees of TH protein and dopamine loss associated with the same magnitude of GTPCH protein, BP, and NP loss in the putamen between the symptomatic and asymptomatic GTPCH-deficient DRD cases suggest that 1) there are additional genetic and/or environmental factors modulating the regulatory effect of BH_4_ on TH protein stability in the striatum and 2) the extent of striatal protein reduction in TH, rather than that in GTPCH, may be critical in determining the symptomatic state of GTPCH-deficient DRD and could contribute to gender-related incomplete penetrance of *GCH1* mutations in this treatable disorder.

## Funding

No funding was received.

## Author contributions

YF wrote the manuscript and approved the final version.

## Conflicts of interest statement

The author declare that there are no conflicts of interest.
